# Correction: Stressed-out yeast do not pass GO

**DOI:** 10.1083/jcb.20211103201052022c

**Published:** 2022-01-21

**Authors:** Hilary A. Coller

Vol. 221, No. 1 | 10.1083/jcb.202111032 | December 21, 2021

After publication, an error was brought to the author's attention. The yeast protein XBP1 had been described as "an ER stress response–induced" transcription factor; however, the Xbp1 protein in yeast is not associated with the ER stress response, and it is a different protein from XBP1 in mammalian cells. "ER stress" was subsequently removed from the text in three instances, and the corrected sentences appear as follows:

"They discover that cells arrest not only in the early G1 phase as expected, but also later in the cell cycle, and that Xbp1 is critical for arrest at other cell cycle phases."

"In contrast, the accumulation of nuclear Sfp1, a regulator of ribosomal protein gene expression, Gln3, a nitrogen stress transcription factor, and Xbp1, a stress-induced histone deacetylase regulator/transcriptional repressor, did differ between the two cell groups."

"The authors’ finding that stress regulator Xbp1 determines how cells respond to starvation—by arresting with a low or high CDK state—supports the existence of a molecular system that can override cell cycle control by CDKs."

These changes do not alter the conclusions of the Spotlight. The errors appear only in print and in PDF versions downloaded on or before January 4, 2021.

